# Multi‐omics integration reveals a core network involved in host defence and hyperkeratinization in psoriasis

**DOI:** 10.1002/ctm2.976

**Published:** 2022-12-19

**Authors:** Jingwen Deng, Emmerik Leijten, Michel Olde Nordkamp, Guangjuan Zheng, Juliëtte Pouw, Weiyang Tao, Sarita Hartgring, Deepak Balak, Rianne Rijken, Runyue Huang, Timothy Radstake, Chuanjian Lu, Aridaman Pandit

**Affiliations:** ^1^ Guangdong Provincial Hospital of Chinese Medicine Guangzhou University of Chinese Medicine Guangzhou China; ^2^ Center for Translational Immunology University Medical Center Utrecht, Utrecht University Utrecht The Netherlands; ^3^ Department of Rheumatology and Clinical Immunology University Medical Center Utrecht, Utrecht University Utrecht The Netherlands; ^4^ Department of Dermatology LangeLand Hospital Zoetermeer The Netherlands

**Keywords:** gene network, hyperkeratinization, multi‐omics, psoriasis

## Abstract

**Objectives:**

The precise pathogenesis of psoriasis remains incompletely explored. We aimed to better understand the underlying mechanisms of psoriasis, using a systems biology approach based on transcriptomics and microbiome profiling.

**Methods:**

We collected the skin tissue biopsies and swabs in both lesional and non‐lesional skin of 13 patients with psoriasis, 15 patients with psoriatic arthritis and healthy skin from 12 patients with ankylosing spondylitis. To study the similarities and differences in the molecular profiles between these three conditions, and the associations between the host defence and microbiota composition, we performed high‐throughput RNA‐sequencing to quantify the gene expression profile in tissues. The metagenomic composition of 16S on local skin sites was quantified by clustering amplicon sequences and counted into operational taxonomic units. We further analysed associations between the transcriptome and microbiome profiling.

**Results:**

We found that lesional and non‐lesional samples were remarkably different in terms of their transcriptome profiles. The functional annotation of differentially expressed genes showed a major enrichment in *neutrophil activation*. By using co‐expression gene networks, we identified a gene module that was associated with local psoriasis severity at the site of biopsy. From this module, we found a ‘core’ set of genes that was functionally involved in *neutrophil activation*, *epidermal cell differentiation* and *response to bacteria*. Skin microbiome analysis revealed that the abundances of *Enhydrobacter*, *Micrococcus* and *Leptotrichia* were significantly correlated with the genes in *core network*.

**Conclusions:**

We identified a core gene network that associated with local disease severity and microbiome composition, involved in the inflammation and hyperkeratinization in psoriatic skin.

## Graphical abstract

- There were dramatic differences between molecular profiles of lesional and non‐lesional skin.
- A core network of genes is strongly associated with local disease severity, and it involved in the regulations of inflammation and hyperkeratinization in psoriatic lesion.
- Microbial imbalance in skin environment is identified in psoriatic skin, and it is associated with the core network of genes.

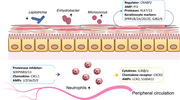

## 1 INTRODUCTION

Psoriasis is a chronic inflammatory skin disease which is characterized by erythematous plaques covered with silvery scales. Psoriasis affects ∼125 million people globally and has multiple comorbidities associated with it, including psoriatic arthritis (PsA).^1^, ^2^ Psoriasis is a complex disease. The development and progression of psoriasis has been associated with several genetic, environmental and immune factors. Abnormal differentiation of keratinocytes and excessive immune cell infiltration in the skin is considered to be the primary aetiology of psoriasis.^3^

Extensive evidence implicates the crucial role T cells play in psoriasis.^4^ However, recent investigations demonstrated that patients with psoriasis have innate immunity disorders, which may have a pivotal impact in the pathogenesis of psoriasis.^5^, ^6^, ^7^ In these studies, the dysfunction of neutrophils, dendritic cells and other components of the innate immune system have been reported in patients with psoriasis. Proinflammatory elements activate neutrophils that secrete proinflammatory cytokines (IL‐6, TNFα).^8^ Dendritic cells are also stimulated, resulting in IL‐23/IL‐12 release, which activates Th17 cells.^9^ Other innate immune cells, such as group 3 innate lymphoid cells, γδ T cells, natural killer cells and natural killer T cells, appear to contribute to the pathogenesis of psoriasis by producing IL‐17.^10^, ^11^ However, the precise molecular mechanism underlying innate immunity disturbance and hyperkeratinization has remained inconclusive.

Increasing evidences suggest that innate immunity through host–microbe interactions is a critical link in the pathogenesis of psoriasis.^8^, ^12^ The skin is a physical protective barrier and dynamic interface for host–microbe interactions. The communication between commensal skin microbiome and host defence may affect the development of psoriasis. For instance, *Staphylococcus aureus* and *Staphylococcus epidermidis* have been reported to induce the expression of antimicrobial peptides (AMPs), leading to the activation of skin innate immune system.^13^ The increased diversity and low community stability of microbiome in psoriatic skin has been established, potentially exacerbating inflammation along the Th17 axis.^14^ However, with single‐layer omics techniques, the broad‐spectrum interplay of host and microbiota is lacking in these studies.

In this study, we integrated multi‐level data from transcriptome and microbiome based on a cohort of patients with psoriasis and PsA. Our goal was to achieve a better understanding of the underlying mechanisms of psoriasis. Moreover, some of these proposed signatures may be potential to serve for diagnostics, prognostics and therapeutic targets of psoriasis.

## 2 MATERIALS AND METHODS

### 2.1 Study design

This study was conducted at the University Medical Centre Utrecht (UMCU) and performed in compliance with the Helsinki principles. Skin biopsies, skin swabs and clinical parameters were collected from a cohort of patients with psoriasis, PsA (fulfilled ClASsification of Psoriatic ARthritis criteria)^15^ and ankylosing spondylitis (AS) (fulfilled Assessment of SpondyloArthritis international Society criteria)^16^ in a prospective observational study (*n* = 40 patients). None of the patients with AS had a history of psoriasis. The recruitment of participants was performed at the outpatient clinic of the Department of Rheumatology and Clinical Immunology in UMCU from March 2016 to September 2018.

Skin samples were derived from 4‐mm punch full‐depth biopsies that were embedded in Tissue‐Tek and directly snap‐frozen in liquid nitrogen, until further processing. Skin microbiota samples were collected by rubbing sterile swabs submerged in SCF‐1 buffer for 1 min on lesional or non‐lesional psoriatic skin sites. After swabbing, the swab tips were stored in cryovials and stored at −80°C.

### 2.2 Evaluation of psoriasis severity at the site of biopsy

The measurement for local disease activity was the Psoriasis Severity Index (PSI).^17^ The Psoriasis Area Severity Index (PASI) scoring method was performed to give a PSI score for the site where biopsy/swab of affected skin was performed (cumulative score of 0–12 based on the total sum: (0–4 redness) + (0–4 thickness) + (0–4 scaling)).

### 2.3 RNA‐Seq analysis

Skin samples were cut (20 µm) and dissolved and lysed in RLT plus (+BME) buffer. Samples were vortexed and homogenized by pipets before RNA isolation. RNA isolation was performed using a QIAGEN Universal Kit, according to manufacturer's protocol. Samples were then prepared according to standard operating procedures of GenomeScan BV for sequencing. RNA sequencing (polyA enriched) was performed using Illumina NovaSeq 6000 Sequencing platform (paired‐end, 150 bp) resulting in ∼6 Gb (20 million paired‐end reads) per sample of Illumina‐filtered sequence data.

The reads obtained were aligned to human genome (GRCh38 build 99) using STAR aligner (version 020201), and mapped reads were counted using HTSeq‐count (version 0.9.0).^18^, ^19^ For all the samples, >80% reads unambiguously mapped to the human genome and of those >80% aligned to the annotated genes. DESeq2 (version 1.32.0) was used for normalization and differential analysis.^20^ In differential expression analysis, the likelihood ratio test was applied for multivariable or pairwise comparisons.

### 2.4 Functional annotation

Gene ontology (GO) and Reactome pathway analysis were conducted to identify the biological function of the gene sets. Gene set enrichment analyses (GSEAs) were performed to calculate an enrichment score of gene profiles according to ontology gene sets in the Molecular Signatures Database C5 collection.^21^, ^22^ Normalized enrichment score (NES) was calculated to quantify the gene enrichment. A positive NES indicates the enrichment of genes at the top of the ranked molecular signatures, whereas a negative NES indicates those at the bottom of the ranked list. The NES is helpful to compare the results across different molecular signatures. All these analyses and visualizations were conducted using the R package clusterProfiler (version 4.0).^23^

### 2.5 Estimation of immune cell infiltration

To estimate the immune cell infiltration in skin via gene expression profiles, we applied a web‐based computational deconvolution tool xCell.^24^ A total of 22 functionally defined human immune cell types (Charoentong signatures) were profiled. Gene signature–based enrichment scores of immune cells were calculated in the website of xCell (https://xcell.ucsf.edu/).

### 2.6 Skin biopsy histological analysis

The skin biopsy obtained from a patient with psoriasis was fixed in 4% (w/v) formaldehyde solution for 24 h. After the fixation, it was embedded in paraffin and then cut into slides. We stained the slides with haematoxylin and eosin.

### 2.7 Weighted gene co‐expression network analysis (WGCNA)

Filtered log2CPM normalized gene expression data of lesional samples were used as input for a biweight‐midcorrelation‐signed network constructed by weighted gene co‐expression network analysis (WGCNA) package (version 1.70.3) in R.^25^ This gene co‐expression network was constructed with the following parameters: maxBlockSize = 20 000, soft power threshold = 7, minModuleSize = 30, mergeCutHeight = .2, corType = ‘bicor’ and networkType = ‘signed’. Network visualization was performed with Cytoscape software (version 3.7.0).^26^

The correlation between individual gene expression and clinical trait was defined as the gene significance (GS). The correlation between individual gene expression and the module eigengene of a given module was defined as the module membership (MM).

### 2.8 Gene regulator‐target network analysis

Gene regulator‐target network for the transcription factors was constructed by using the random forest method with RegEnrich R package (version 1.3.0).^27^ We analysed the regulator–target relationship between transcription factors provided by RegEnrich and the differentially expressed genes (DEGs) obtained from the psoriatic lesional and non‐lesional comparison. RegEnrich provided us ranked list of the regulators along with their significance calculated by integrating the differential expression of regulators, their targets and enrichment analysis. In gene regulatory network inferring, both Fisher's exact test and GSEA were used for the enrichment test.

### 2.9 *Core network* signature analysis

Gene set variation analysis (GSVA) was used to determine the entirety expression level of *core network*.^28^ By comparing the GSVA scoring of *core network*, we estimated the variation in the gene enrichments of the *core network* across different independent cohorts. The NES of *core network* over the psoriatic lesions and controls was measured with R package GSVA (version 3.11). NES ranged from −1 to 1, with negative scores indicating relative decreases in network expression, whereas positive scores indicated elevations.

### 2.10 Validation of *core network* with public datasets

Seven public datasets (E‐MTAB‐8142, GSE13355, GSE41745, GSE63979, GSE67785, GSE83645 and GSE54456) for psoriasis transcriptome studies were utilized for the validation of *core network* with their GSVA scoring of *core network*.^29^, ^30^, ^31^, ^32^, ^33^, ^34^, ^35^ The criteria for bulk RNA‐Seq datasets selection are (a) the dataset had been already pre‐processed, harmonized and prepared according to the FAIR principles^36^, ^37^; (b) the comparison was lesion versus non‐lesion and (c) datasets with at least three samples in each group. Among these seven datasets, one dataset (E‐MTAB‐8142) was detected with single‐cell sequencing, one (GSE13355) was performed with bulk microarray and the other five were performed with bulk RNA‐Seq. In our re‐analysis, normal skin from healthy donors was used as control for one dataset (GSE54456). For other six datasets, non‐lesional skin from same cohort was applied as control (Table S3). Volcano plots for DEGs profile across independent cohorts were visualized with R package EnhancedVolcano (version 1.12). Heat maps for correlation of genes in specific cell‐types were visualized with R package corrplot (version 0.92).^39^

### 2.11 Single‐cell transcriptome analysis

Single‐cell dataset was obtained from ArrayExpress database (E‐MTAB‐8142).^35^ Data normalization, scale, highly variable genes selection, principal components calculation and dimensionality reduction were performed using Scanpy (version 1.8.2).^39^ Highly variable genes were detected with minimum cut‐off values .0125 and .5 for expression and dispersion, respectively. Batch correction (donor‐to‐donor variation) was adjusted using the BBKNN package (version 1.5.1).^40^ Cell‐type annotation was based on gene expression profiles provided by source data. All these analyses were performed in Python (version 3.8.10).^41^

### 2.12 16S sequencing

Skin swab DNA extraction was performed using the QIAamp UCP Pathogen Mini Kit automated on the QIAcube. The skin swab was transferred to a tube filled with .65‐ml ATL buffer and incubated for 10 min, 56°C with continuous shaking at 600 rpm. Subsequently, 40‐µl Proteinase K was added, and bead beating was performed using the SpeedMill PLUS for 45 s, at 50 Hz. Samples were then processed according to the manufacturer's protocol. Approximate amounts of DNA were 1–10‐µg/µl per sample. Variable regions V1 and V2 of the 16S rRNA gene were amplified using the primer pair 27F‐338R (27F = AGAGTTTGATCCTGGCTCAG; 338R = TGCTGCCTCCCGTAGGAGT) in a dual‐barcoding approach.^43^, ^44^, ^45^, ^46^ DNA was diluted 1:10 prior PCR, and 3 µl of this dilution was used for amplification. PCR was performed based on the protocol described by Costello et al.^47^ PCR‐products were verified using electrophoresis in agarose gel. PCR products are normalized using the SequalPrep Normalization Plate Kit (Thermo Fisher Scientific) and pooled equimolarly. 16S Sequencing of the skin swabs was done using Illumina MiSeq v3 (2 × 300 bp) platform. Demultiplexing after sequencing is based on 0 mismatches in the barcode sequences. An average of 37.5 thousand reads per sample was obtained for the downstream analysis.

### 2.13 Skin microbiota data analysis

Considering the 16S rRNA data may fail to provide sufficient resolution and accuracy to adequately perform species‐level analysis; in our study, only genus‐level taxonomy was used for the downstream analysis. To preserve statistical power, only operational taxonomic units (OTUs) presented more than 5 reads in at least 20% samples were retained. A total of 140 OTUs in genus level was kept for downstream analysis after the prefiltering. Abundances of microbiota were transformed using the centred log‐ratio method. Principal coordinates analysis (PCoA) based on the Jensen–Shannon divergence was performed to visualize differences in the bacterial community structure across samples. Shannon diversity index of microbiota was calculated by vegan packages (version 2.5‐5). Microbial abundance was compared across different phenotypes with permutational multivariate analysis of variance. Multivariable association between microbiota and phenotypes was analysed by MaAsLin2 (version 3.13).^47^ The circus plot for microbiota–gene association was made by Circos (http://circos.ca/).

### 2.14 Statistical analysis

For clinical characteristics, the chi‐square test was applied for non‐continuous variables, and analyses of variance were applied for the continuous variables. For multiple hypothesis testings, *p* values were adjusted with the Benjamini–Hochberg method, and adjusted false discovery rate (FDR) *p* values < .05 were the threshold for significant difference. Spearman correlation was calculated between gene expression and microbial abundance. All these statistical analyses were performed using stats package (version 4.1.2) in R (version 4.0.3) (http://cran.r‐project.org/).

## 3 RESULTS

### 3.1 Cohort description

The cohort included 13 psoriasis patients with a dermatologist‐confirmed diagnosis of psoriasis in whom concomitant PsA was clinically excluded by a rheumatologist; 15 patients with PsA and 12 patients with AS were included as a non‐psoriatic reference group, none of which had a history of psoriasis.

The psoriasis, PsA and AS groups were matched for age, gender, body mass index and smoking. The disease duration for psoriasis, PASI and PSI scores were similar between the psoriasis and PsA groups. Erythrocyte sedimentation rate and C‐reactive protein, as two biomarkers commonly used to detect inflammation, showed no difference between diseases. The clinical characteristics of the participants are shown in Table 1.

**TABLE 1:** Clinical characteristics of the participants

	Psoriasis (*n* = 13)	PsA (*n* = 15)	AS (*n* = 12)	*p* Value
Sex				
Female	7 (53.8%)	5 (33.3%)	3 (25.0%)	.302
Male	6 (46.2%)	10 (66.7%)	9 (75.0%)	
Age (years)	41.3 (13.2)	46.3 (9.32)	44.2 (8.54)	.466
BMI (kg/m^2^)	28.2 (7.79)	26.9 (4.62)	25.2 (2.66)	.728
Smoking				
Never	3 (23.1%)	4 (26.7%)	5 (41.7%)	.509
Past	5 (38.5%)	7 (46.7%)	6 (50.0%)	
Present	5 (38.5%)	4 (26.7%)	1 (8.3%)	
Psoriasis duration (years)	24.2 (14.6)	23.1 (16.1)	–	.734
PASI	6.83 (5.40)	5.05 (4.87)	–	.249
Local PASI	5.31 (2.56)	5.54 (2.15)	–	.677
DMARD history				
Yes	1 (7.7%)	7 (46.7%)	2 (16.7%)	.0433^*^
No	12 (92.3%)	8 (53.3%)	10 (83.3%)	
UVB history				
Yes	7 (53.8%)	3 (20.0%)	–	.0623
No	6 (46.2%)	12 (80.0%)	–	
Biologic history				
Yes	12 (92.3%)	14 (93.3%)	–	.916
No	1 (7.7%)	1 (6.7%)	–	
ESR (mm/h)	9.46 (12.9)	8.27 (9.61)	7.82 (7.78)	.922
CRP (mg/L)	4.97 (7.30)	5.04 (6.85)	4.94 (5.22)	.955

*Note*: Means (standard deviation) are presented for continuous values. Frequencies (proportion) are presented for categorical values. DMARD, UVB or biologic history: usage in past 3 months.

Abbreviations: AS, ankylosing spondylitis; BMI, body mass index; CRP, C‐reactive protein; DMARD, disease‐modifying antirheumatic drug; ESR, erythrocyte sedimentation rate; PASI, Psoriasis Area Severity Index; PsA, psoriatic arthritis.

* Significant at *p* value <.05.

### 3.2 Transcriptome profile significantly altered between psoriatic lesional and non‐lesional skin

**FIGURE 1:**
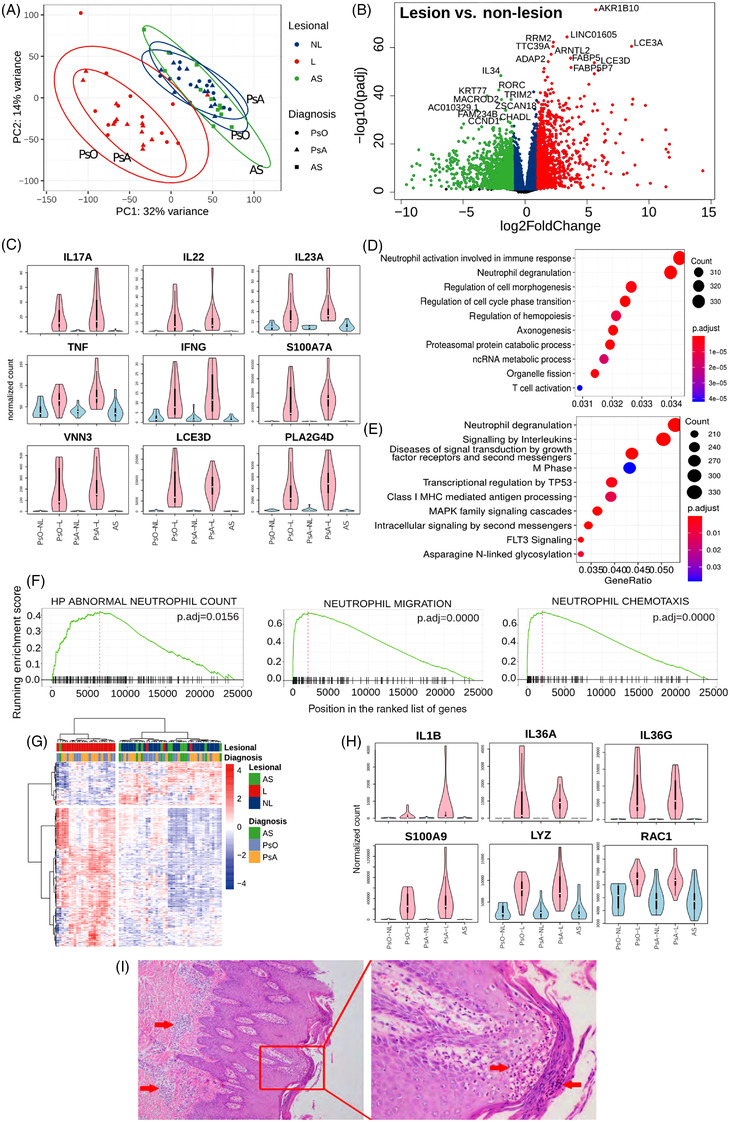
Differential expression profile in skin transcriptome: (A) principal component analysis (PCA) of all samples; (B) volcano plot of differentially expressed genes (DEGs) in psoriatic lesion compared to non‐lesion. The red dots are the genes with both FDR smaller than .05 and log2FoldChange greater than 1. The green dots are the genes with both FDR smaller than .05 and log2FoldChange smaller than −1; (C) expression profile of psoriasis markers DEGs. Gene expression values were normalized with median of ratios method; (D) top 10 gene ontology (GO) annotations for the DEGs; (E) top 10 Reactome annotations for the DEGs; (F) gene set enrichment analysis (GSEA) of the gene expression profile for the enrichment of neutrophil counts and function; (G) heat map of the gene expression profile of neutrophil activation; (H) expression profile of neutrophil markers; (I) histological section of psoriatic lesion (left: 10×; right 40×). Haematoxylin and eosin (H&E) staining demonstrates an accumulation of neutrophils in the skin.

We first examined the differences between conditions (psoriasis, PsA and AS) and type of skin samples (lesional and non‐lesional). The principal component analysis found that the lesions from psoriasis overlaid with the lesions from PsA, whereas all the non‐lesional samples clustered together (Figure 1A). Differential expression analysis showed that lesions did not differ between conditions (psoriasis and PsA). Similarly, we found minimal differences among non‐lesional samples based on their conditions (0 DEG for psoriasis vs. PsA, and 2 DEGs for psoriasis+PsA vs. AS comparisons) (Table 2).

**TABLE 2:** Results of differential expression analyses on different comparisons

Comparison	Pairwise	DEGs (FDR < .05)		
		Total	Upregulated	Downregulated
Lesions from Pso and PsA vs. non‐lesions from Pso and PsA	Yes	13 700	6093	7607
Lesions from Pso and PsA vs. non‐lesions from Pso, PsA and AS	No	13 205	5949	7256
Pso lesion vs. Pso non‐lesion	Yes	7916	3836	4080
PsA lesion vs. PsA non‐lesion	Yes	9983	4907	5076
Pso lesion vs. PsA lesion	No	0	0	0
All non‐lesion vs. AS	No	2	1	1
Non‐lesional Pso vs. non‐lesional PsA	No	0	0	0

Abbreviations: AS, ankylosing spondylitis; DEG, differentially expressed gene; PsA, psoriatic arthritis.

As we did not find differences between types of samples from different patient cohorts, we pooled the psoriatic diseases together for the differential expression analysis to further study the aetiology of psoriatic lesions compared to non‐lesional skin. We found remarkably a high number of DEGs (13 700 genes; 6093 increased and 7607 decreased) in pairwise analysis of the lesional samples with the non‐lesional samples from psoriasis and PsA (Tables 2 and S1). The commonly used markers of psoriasis were significantly increased in lesions compared to non‐lesional samples^9^, ^49^ (Figure 1C). Most of the DEGs we found were consistently increased in lesions of psoriasis and PsA patient when compared to non‐lesional skin samples from psoriasis, PsA and AS (Figure S1A).

To understand the remarkable difference between the transcriptome profiles of lesional and non‐lesional samples, we next performed the deconvolution analysis to predict the cell compositions of the different skin samples. In line with previous literatures, the different subsets of T cells (bulk CD4+, bulk CD8+, effector memory CD8+ and regulatory T cells) were more abundant in lesional skin compared to non‐lesional skin (Figure S2). These observations suggested a large immune infiltration in the skin microenvironment.

### 3.3 Neutrophil‐mediated inflammation in psoriatic lesion

To understand the functional implications of the changes in transcriptomic profiles of lesional skin, we next performed functional annotation analysis (using GO, Reactome pathways and GSEA). We found that genes attributed to GO terms *neutrophil activation involved in immune response* (GO:0002283) and *neutrophil degranulation* (GO:0043312) were significantly enriched in lesional skin (Figure 1D). Reactome pathways analysis further supported neutrophil involvement in psoriasis, as *neutrophil degranulation* was the top one pathway enriched in lesional skin (Figure 1E). Using the expression profile of 335 DEGs constituting the GO term of *neutrophil activation involved in immune response* clearly separated the lesions from the non‐lesional and AS samples (Figure 1G). Among of these 335 DEGs, more than 75% of these genes associated with functions of neutrophil were upregulated in lesions, including neutrophil activation makers (IL1β, IL36A, IL36G), chemotaxis maker (S100A9), defencing maker (LYZ) and migration maker (RAC1) (Figure 1H).^49^ When validated these neutrophil activation makers and the markers of psoriasis in Figure 1C with the public datasets, we found that S100A7A, LCE3D, PLA2G4D, LCN2, IL36G and S100A9 were consistently significantly increased in lesional group (Figure S3).

The GSEA further corroborated that the differences in gene expression profile in lesional skin compared to non‐lesional skin were linked to the upregulation of the cell counts and function of neutrophils. We found significant enrichments of *abnormal neutrophil count*, *neutrophil migration* and *neutrophil chemotaxis* (Figure 1F). Using the deconvolution analysis, we confirmed that neutrophils were the only myeloid cells that increased in their abundance in psoriatic lesions (Figure S2). In summary, multiple analyses indicate that neutrophils are dysregulated in psoriatic lesions, especially in their abundance, migration and chemotaxis.

### 3.4 Transcriptome alterations were associated with the severity of local lesion rather than the global severity

**FIGURE 2:**
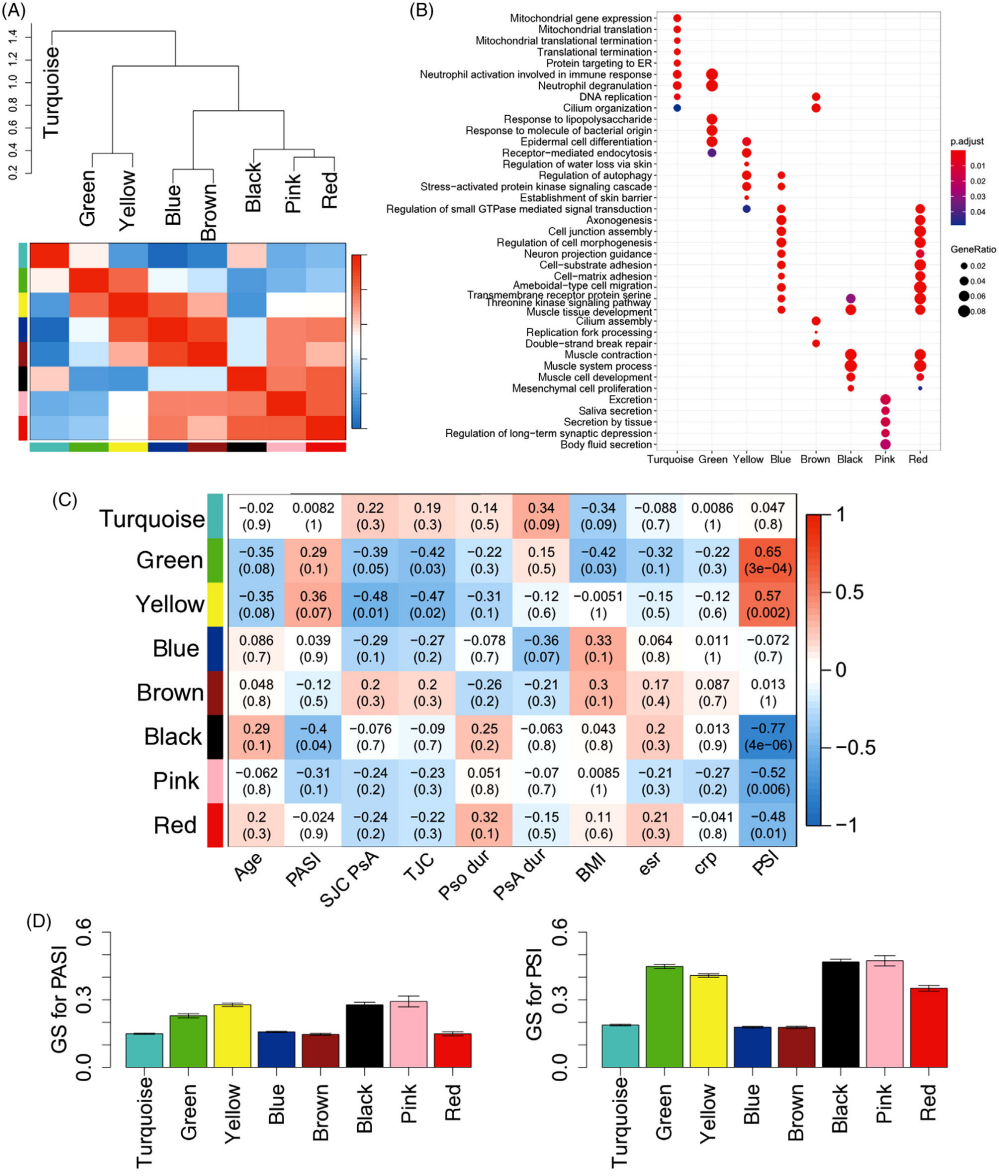
Weighted gene co‐expression network analysis (WGCNA) construction for gene expression profile and module–trait relationship in lesional samples: (A) hierarchical clustering tree (dendrogram) and gene co‐expression module definition in lesional samples. A total of eight modules were identified; (B) gene ontology (GO) annotation for the modules; (C) table of module–trait (clinical parameter) correlations and *p* values. Each cell reports the correlation (and *p* value) resulting from correlating module eigengenes (rows) to traits (columns). The table is colour‐coded by correlation according to the colour legend; (D) the significant relationship between modules and phenotypic variables Psoriasis Area Severity Index (PASI) score and PSI score. The results are expressed as the means ± SD. GS, gene significance

To obtain clusters of genes (modules) involved in common functions and related to the clinical phenotypes, we constructed the co‐expressed network with the gene expression profile of lesional samples using WGCNA. Within this lesional network, we identified eight co‐expression modules (black, blue, brown, green, pink, red, turquoise and yellow) (Figure 2A). We annotated these eight modules with GO terms and correlated them with clinical parameters (Figure 2B,C). We found that disease severity was correlated with the eigengene of five modules (green, yellow, black, pink and red modules; Figure 2C). Typically, the severity of lesion where we took the biopsies (PSI) was more correlated with the gene modules as compared to the PASI (Figure 2D).

### 3.5 *Core network* was found to correlate with disease severity

We next focused on the PSI‐associated modules (green, yellow, black, pink and red). Green and yellow modules were positively correlated, whereas the other three were negatively correlated with the PSI (Figure 2C). To assess if a gene is central or hub of the module and hence important from gene network, we further investigated the GS of each gene in green and yellow modules by correlating their expression to PSI, and their MMs (Figure S4C,D). The hub genes in green module had higher positive correlations and hence were coupled to the PSI better than those in yellow module. Moreover, green module was enriched in genes associated with psoriasis‐related functional pathways, such as *epidermal cell differentiation*, *the neutrophils activation*, *neutrophils degranulation*, *receptor‐mediated endocytosis*, and *response to LPS and bacteria* (Figure S4E); however, yellow module genes were enriched in skin barrier–related functional pathways.

**FIGURE 3:**
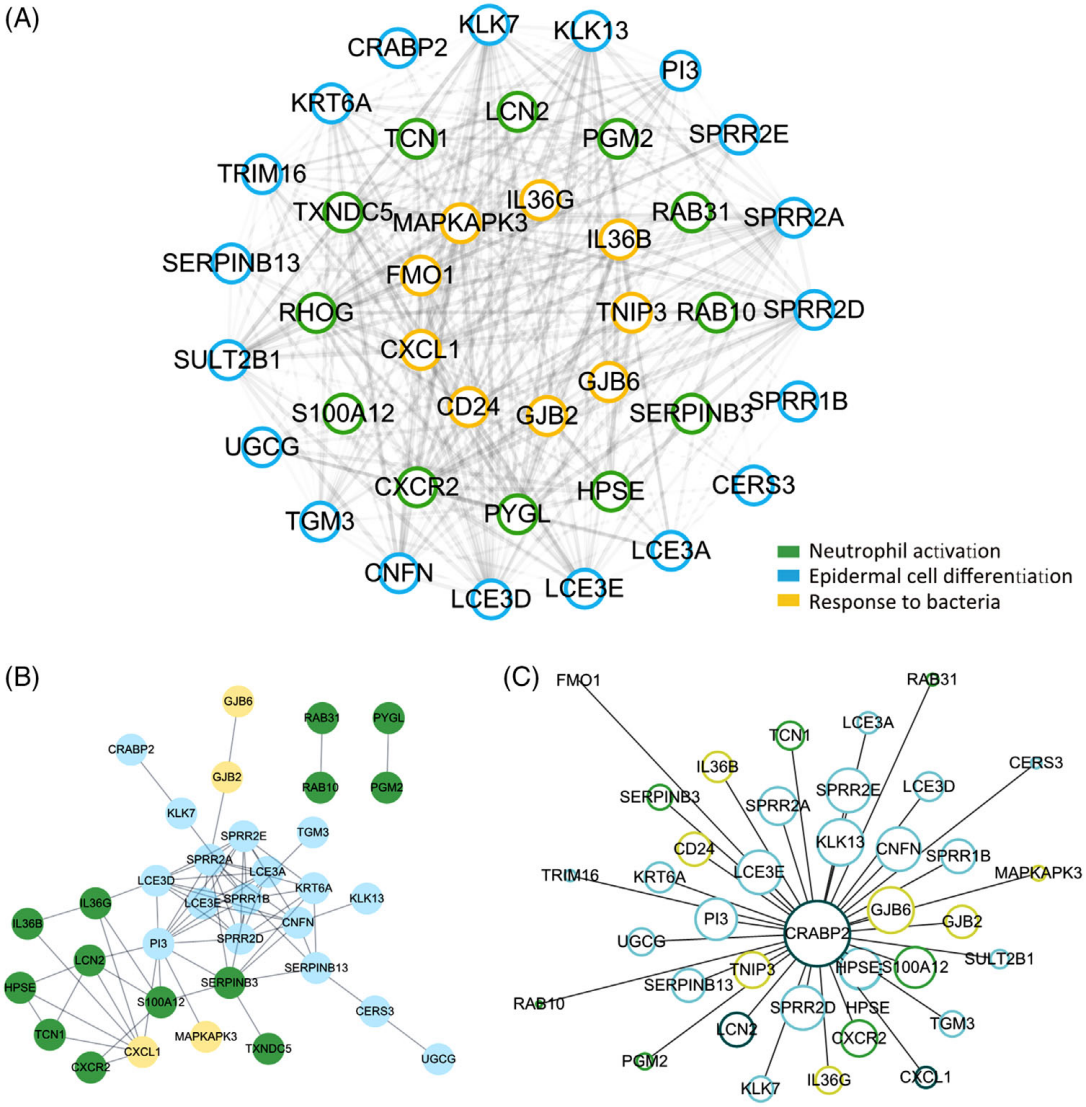
Core network exploration in green module: (A) core network in green module: The clusters with the highest adjacency in heat map (two triangles) were extracted and visualized with cytoscape. Genes for neutrophil activation (light blue), epidermal cell differentiation (green) and response to bacteria (yellow); (B) protein‐protein interactions network for core network genes; (C) gene regulatory network of CRABP2 and its target genes only showed the genes overlap with core network. Gene regulatory analysis was based on random forest algorithm.

We then integrated the knowledge about functionality of the genes from literature and importance of the genes as derived from our data‐driven networks. To do so, we extracted the genes that were hub genes in the green gene module and were functionally annotated in psoriasis related GO terms (*epidermal cell differentiation*, *neutrophils activation and degranulation* and *response to LPS and bacteria*). By measuring the adjacencies as the edge weights, these hub genes were functionally related and were highly correlated with each other forming a ‘*core network*’ of genes (Figure 3A).

We considered that the genes in the *core network* could be highly correlated with each other as (a) they are physically interacting with each other, or (b) they are regulated by the same set of transcriptional regulators. So, we first explored if the genes in the *core network* are known to have physical interactions using the STRING database^51^ and found that 75% of the *core network* genes were involved in physical protein–protein interactions (Figure 3B). We further used RegEnrich to perform the gene regulatory network analysis. A large proportion (36/40) of genes in *core network* were overlapped with the target genes of CRABP2 in gene regulatory network, which implied that the genes in *core network* not only represented co‐expression pattern but also the regulator‐target relationship (Figure 3C). Interestingly, both protein–protein network analysis and gene regulatory network analysis revealed that the *core network* gene CRABP2, a carrier protein for retinoic acid signalling pathways, is an important regulator of genes within the *core network* and of genes differential in lesional samples (Figures 3B,C and S5A). All these evidences indicated that the genes in *core network* had more interactions among themselves than what would be expected for a random set of genes, and that these genes were at least partially biologically connected to each other as a network.

### 3.6 *Core network* was validated across independent cohorts

**FIGURE 4:**
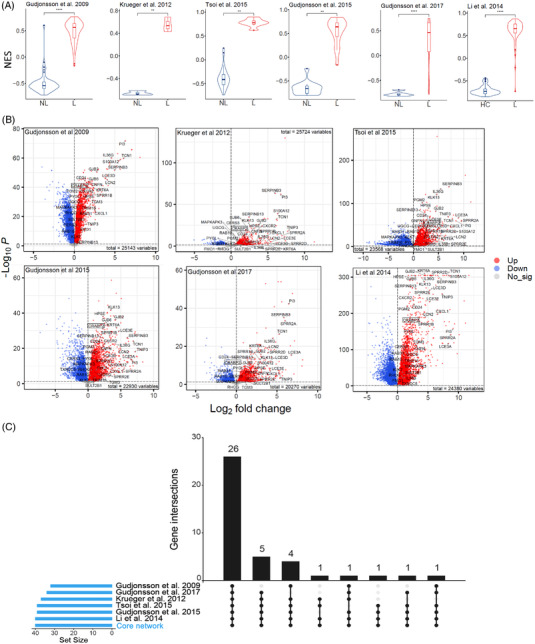
Validation of *core network* signature with public datasets: (A) normalized enrichment score (NES) of *core network* across six independent bulk RNA‐Seq datasets. *p < .05, **p < 0.01, ****p < 0.0001; (B) volcano plots of six independent bulk RNA‐Seq datasets. The red dots are the genes with both FDR smaller than .05 and log2FoldChange greater than 0. The green dots are the genes with both FDR smaller than .05 and log2FoldChange smaller than 0. The genes in *core network* were labelled with their names when they were significantly upregulated; (C) upset plot visualized intersections of genes in *core network* as differentially expressed genes (DEGs) across six independent bulk RNA‐Seq datasets

To ascertain the reproducibility of our *core network*, we used seven independent cohorts of psoriatic skin samples from publicly available datasets.^29^, ^30^, ^31^, ^32^, ^33^, ^34^, ^35^ The information for these seven independent cohorts is listed in Table S3. We used the GSVA method to estimate the strength of *core network* signature in a sample. In this method, a higher score means higher expression of *core network* genes. The *core network* scores of lesional skins were consistently higher than those of the non‐lesional or healthy group across six independent bulk RNA‐Seq datasets (Figure 4A). Using the differential expression analysis, we found that most of genes in *core network* were significantly upregulated in all independent cohorts (Figure 4B). Among these genes, CRABP2 was differentially expressed in lesion across all datasets, with relatively highly fold changes (range of log2FoldChange: 1.81–2.29). A total of 26 genes in *core network* were overlapping as DEGs in different cohorts. There were only few genes not included in DEGs of Gudjonsson's studies (Figure 4C). We considered this difference was caused by the detection platform and study design: one study (Gudjonsson 2009) used microarray (not all the genes were included or detectable in the chips); the other study (Gudjonsson 2017) took four biopsies from different anatomic lesions of same patient. This makes the results difficult to compare with our study as our study and all other studies took one lesional biopsy from one patient.

**FIGURE 5:**
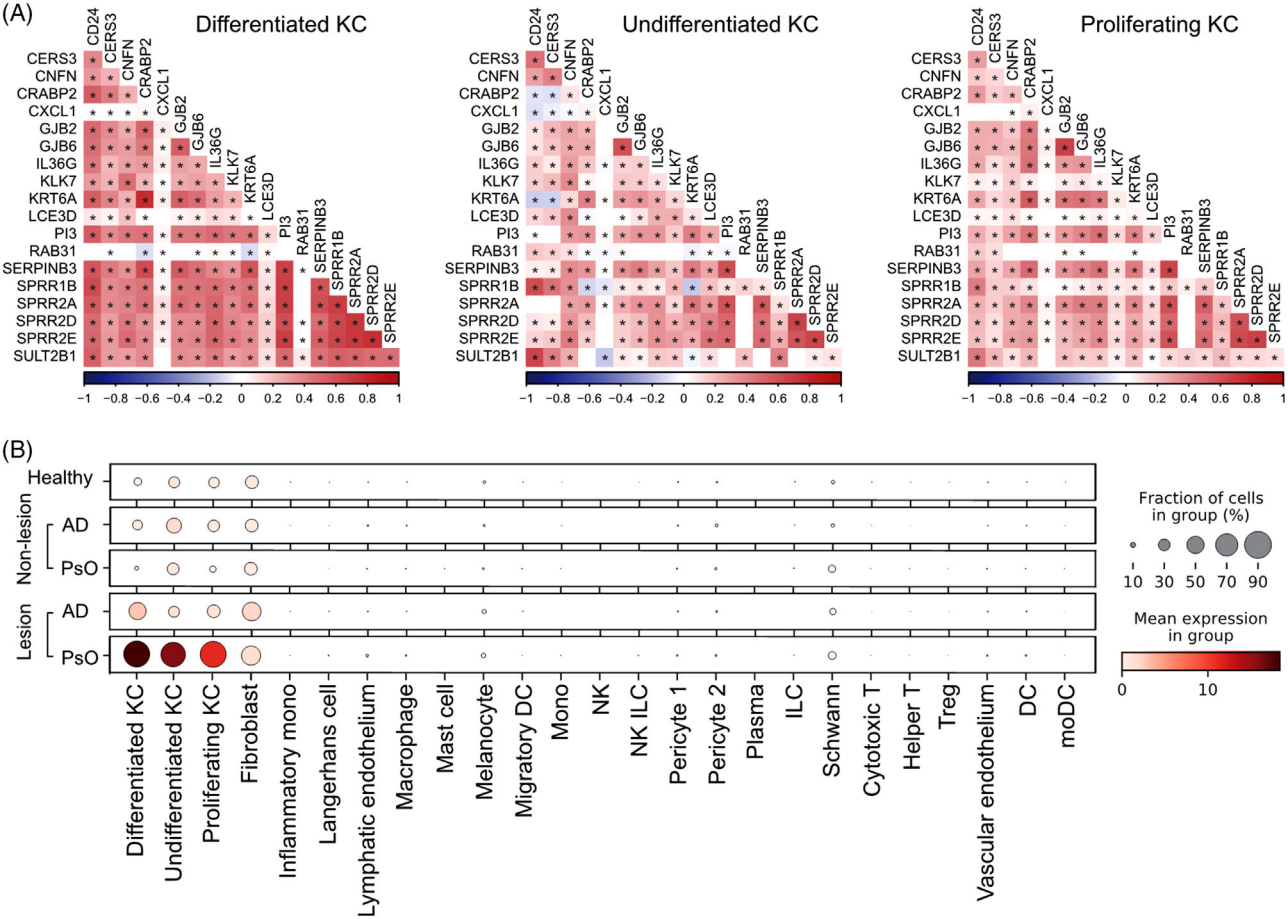
Core network validation in single‐cell level: (A) Heat map of correlation between the genes in *core network* in three subsets of keratinocyte (KC) in a single‐cell dataset of psoriatic skin. * Adjusted *p* < .05; (B) gene expression of CRABP2 in different cell‐types across different conditions. FB, fibroblast; Inf., inflammatory; LC, Langerhans cell; LE, lymphatic endothelium; Mac, macrophage; Mig., migratory; MoDC, monocyte‐derived dendritic cell; Tc, cytotoxic T cell; Th, T helper cell; VE, vascular endothelium

To validate the interaction between the genes in *core network* in specific cell types, we extracted three subsets of keratinocyte (differentiated keratinocyte, undifferentiated keratinocyte and proliferating keratinocyte) from a single‐cell dataset of psoriatic skin. A total of 19 genes in *core network* were detected in keratinocytes. Among these 19 genes, more than 95% of gene–gene correlations were significant positive, especially in differentiated keratinocytes (Figure 5A). Moreover, we found that CRABP2 was highly expressed in lesional skin samples taken from psoriasis patients, lowly expressed in lesional skin samples taken from atopic dermatitis (AD) patients and rarely expressed in healthy skin (Figure 5B). In addition, it was selectively expressed by the three clusters of keratinocytes in psoriatic lesion, whereas differentiated keratinocytes had the highest expression level of CRABP2.

All these evidences indicated that the *core network* we found is consistently and reproducibly highly expressed in psoriatic lesion, with positive associations with each other.

### 3.7 Skin microbiota influenced disease severity and associated with the *core network*

**FIGURE 6:**
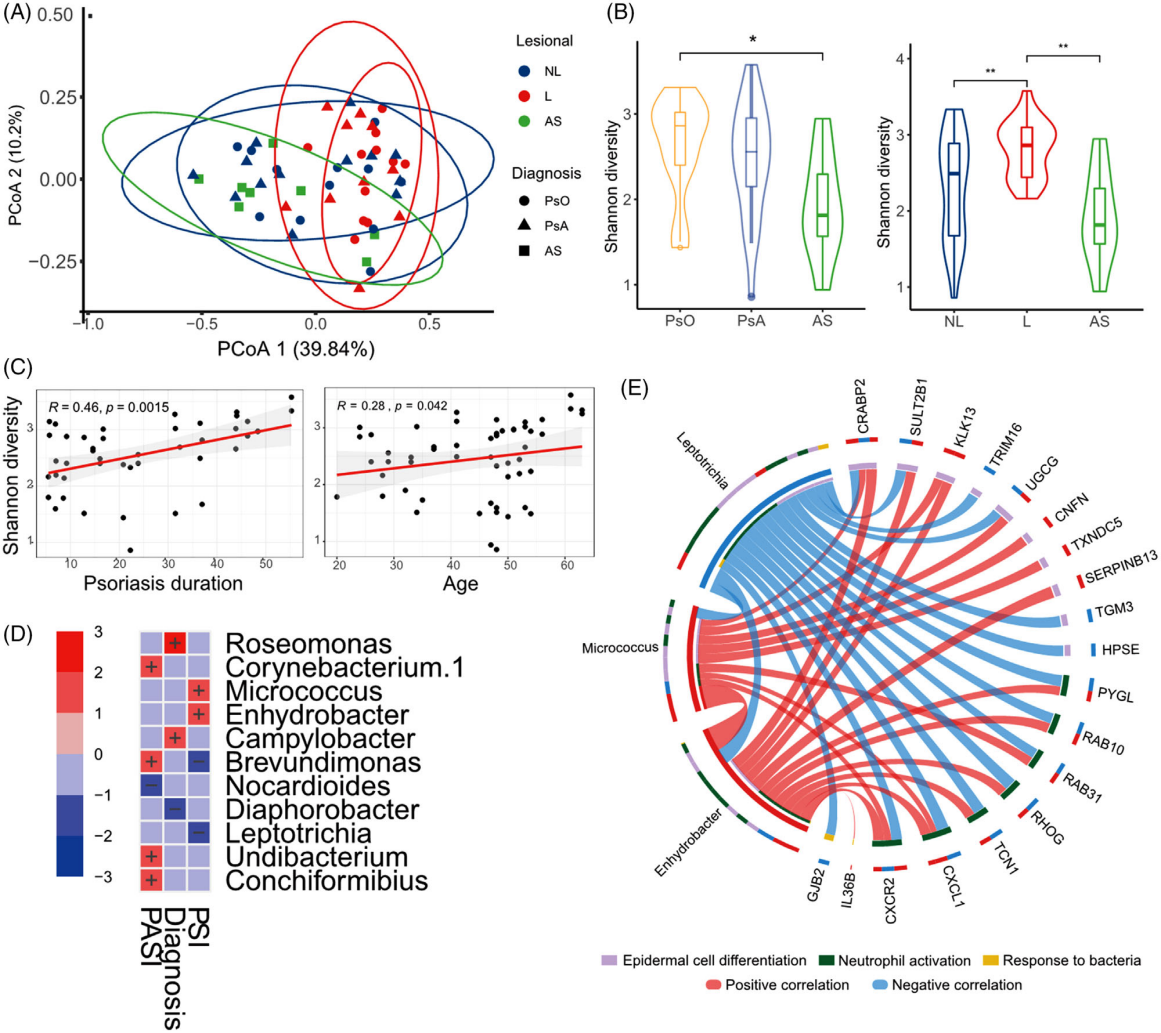
Microbiota profile and host–commensal interaction: (A) principal coordinates analysis (PCoA) of skin microbiota based on operational taxonomic units (OTUs) in genus level; (B) the Shannon diversity of skin microbiota in different cohorts; (C) the association between the Shannon diversity and psoriasis duration (left) or age (right); (D) heat map of the significant associations between skin microbiota abundance and clinical parameters for all samples (upper) and for only lesional samples (lower). Colour for association significance (*p* < .05). Sign ‘+’ and ‘−’ for coefficient; (E) the association between the abundance of *Enhydrobacter*, *Micrococcus*, *Leptotrichia* and *core network* genes. Size of ribbon size encodes *R* values. All ribbons shown are for correlations with statistical significance. *p < .05, **p < .01

As the functional annotation of green module indicated the response to bacteria was involved, we integrated the skin microbiome with the skin transcriptome in our analysis. Similar to the transcriptome profile, PCoA plot showed that the samples were clustered based on skin type (lesional or non‐lesional) and not on clinical phenotype condition (Figure 6A). Psoriasis showed an increase in diversity compared to AS. Lesional pools were more diverse than non‐lesional pools and AS (Figure 6B). The diversity of microbiota in skin was positively correlated with psoriasis duration and age (Figure 6C). To figure out the distinction between lesion and non‐lesion, we additionally carried out a non‐parametric multivariate analysis of variance of clinical phenotypes on the psoriatic skin microbiota. Our results indicated that multiple clinical factors (age, sex, use of disease‐modifying antirheumatic drug etc.) influenced the differential abundance analysis (Table S4). After correction for these confounding effects by using MaAsLin2,^47^ we found three genera differentially enriched between psoriasis and PsA, five genera significantly associated with PASI and four with PSI (Figure 6D).

We subsequently evaluated whether the four PSI‐associated genera were also related to the core gene network. Interestingly, the abundance of *Enhydrobacter*, *Micrococcus* and *Leptotrichia* were strongly correlated with the expression of genes in *core network* (Figure 6E). Specifically, we found CRABP2 was associated with the abundance of all these three genera. Moreover, *Enhydrobacter*, *Micrococcus* and *Leptotrichia* were also significantly correlated with each other (Figure 6E) and implied the potential microbe–microbe interactions in skin.

Overall, novel host gene–microbiome interactions in psoriatic skin were revealed by integrating the profile of disease severity associated genera and gene expression of *core network*.

### 3.8 Drugs targeted to the *core network* was discovered by drug–gene interactions

As we had identified the *core network* and proved its importance in psoriasis, to discover the well‐annotated drug–gene interactions relevant to medical decision‐making based on our *core network*, we further investigated the potential drugs targeted to the *core network* in CLUE database.^52^ The results demonstrated that econazole (antibiotic), methylprednisolone and dexamethasone (glucocorticoid receptor agonist), idalopirdine (serotonin receptor antagonist) among others were targeting the *core network*, implying their potential to be utilized for psoriasis treatments (Tables 3 and S5). Interestingly, these treatments which have been established for psoriasis are also defined as suppressions to the *core network*. Although their application in psoriasis is not novel, the involvement of them supports the reliability of our drug discovery for psoriasis with the *core network*.

**TABLE 3:** Drug targets towards the genes in core network

Drug or component	Mechanism of action
Econazole	Lanosterol demethylase inhibitor; bacterial cell wall synthesis inhibitor; sterol demethylase inhibitor
Methylprednisolone	Glucocorticoid receptor agonist
Idalopirdine	Serotonin receptor antagonist
Nesbuvir	RNA polymerase inhibitor
Rimexolone	Glucocorticoid receptor agonist
Lacosamide	Collapsin response mediator protein stimulant; sodium channel blocker
Reserpic acid	Norepinephrine inhibitor
Dexamethasone	Glucocorticoid receptor agonist; corticosteroid agonist; cytochrome P450 inhibitor
Fursultiamine	Vitamin B
VGX‐1027	TNF inhibitor

## 4 DISCUSSION

High‐throughput profiling techniques (omics) have been widely used to explore the molecular mechanisms in psoriasis. Over the past two decades, several transcriptomics and microbiome profiling studies have established that the host genes as well as the skin microbiota are altered in the psoriatic skin and play a role in the development of psoriasis.^14^, ^29^, ^30^, ^32^, ^33^, ^34^, ^35^, ^53^, ^54^ One multi‐level study integrating transcriptome and microbiome in psoriasis was reported.^55^ Fyhrquist et al. applied gene microarray and 16S sequencing on skin samples of psoriasis and AD. They found that *Corynebacterium* was a regulatory microbe in psoriasis, but the associations between disease related host genes and microbes were weak.^55^ Hence, the broad spectrum of host functions that are mediated by the microbiota remains uncharacterized. The multi‐omics approach we used has identified a *core network* of genes associated with disease severity, inflammation and hyperkeratinization in psoriasis. These *core network* genes were associated with the abundance of skin microbiota, highlighting a direct relationship between the local skin microbiota and the local host transcriptome (see the Graphical Abstract).

The transcription factor CRABP2 may act as a key member in the *core network*. Recent studies have reported that the depletion of CRABP2 reduced viability and proliferation of tumour cells, implied the regulation of CRABP2 in cell proliferation and cell cycle progression.^56^, ^57^ We found that CRABP2 was consistently increased in psoriatic lesion, especially in keratinocytes. We propose that the overexpression of CRABP2 and its downstream pathways are involved in the hyperkeratinization in psoriasis. Furthermore, the expression of CRABP2 was correlated with the disease severity‐associated skin microbes (*Enhydrobacter*, *Micrococcus* and *Leptotrichia*), suggesting the interaction between local environment and immunopathogenesis of the skin.

Apart from CRABP2, many other genes in the *core network* were also strongly correlated with the alterations in the disease severity‐associated skin microbes indicating that microbial imbalances in skin environment is associated with broad changes in the transcriptomic profiles (Figure 6E). These microbial imbalances induce the defensin‐like antimicrobial activity of keratinocytes, including the secretion of AMPs (PI3 and LCE3A/D/E). The defensin‐like antimicrobial activity of keratinocytes has been shown to recruit neutrophils from circulation to skin. For example, overexpression and secretion of CXCL1 by keratinocytes facilitates skin homing of neutrophils by binding to CXCR2 receptor. Activated neutrophils then produce proinflammatory mediators, such as cytokines (IL36β/γ)^57^ and AMPs (LCN2, S100A12), in the skin.^58^ These proinflammatory mediators stimulate the keratinocytes leading to hyperkeratinization ultimately leading to the overexpression of keratinocyte differentiation markers (SPRR1B/2A/2D/2E, GJB2/6)^59^, ^60^, ^61^ and proteases (KLK7/13).^62^

Overall, our results indicate that using a multi‐omics approach we can help identify the core pathways involved in the pathogenesis of psoriasis. A strength of this study is that we captured biological samples (transcriptome, microbiome) from the same anatomical location of the same individual and were able to validate results in publicly available datasets. However, our study had relatively low number of samples. We could detect the main patterns involved in the pathogenesis at the group level with respect to psoriasis versus non‐lesional skin but may not have been able to detect minor aberrances, for example between psoriasis and PsA. Furthermore, as part of the study protocol, we included skin samples from AS patient and have assumed these biopsies to be healthy as these patients did not have clinical skin disease at the site of biopsy nor a history of psoriasis. We cannot exclude the possibility that there are minor changes in non‐lesional psoriasis skin that we have not detected, as reported in some other studies.^64^, ^65^

## 5 CONCLUSION

In summary, we identify a core set of dysregulated genes and specific host–microbe interactions that involve in the pathogenesis of psoriasis. We also identified potential target genes and drugs molecules that can be repurposed to psoriasis but would need further exploration. Further research is needed to determine if modulating the skin microbiota composition and/or therapeutically targeting the *core network* can restore skin homeostasis in psoriasis.

## CONFLICT OF INTEREST

T.R. received consultancy fees from Jansen in 2016 and 2017 on topics that were unrelated to the content of this manuscript. T.R., A.P., and W.T. are currently an employee of AbbVie, with no conflicts of interest regarding the work of this manuscript. D.B. received consultancy fees from Janssen in 2018 and 2019 on topics that were unrelated to the content of this manuscript. The other authors have declared no conflicts of interest.

## FUNDING INFORMATION

This study was financially supported by Janssen Inc and co‐funded by the PPP Allowance made available by Health∼Holland, Top Sector Life Sciences & Health, to stimulate public‐private partnerships. JD was supported by the China Scholarship Council (CSC) NO. 202007720051; National Natural Science Foundation of China (U20A20397, 81603619) and Science and Technology Planning Project of Guangdong Province (2020B1111100005, 2020B1212030006B).

## Supporting information

Figure S1 Venn diagram showed the overlap of DEGs across the L versus NL pairwise comparison and L versus NL versus AS comparison.

Figure S2 Infiltration score of immune cells in skin predicted by deconvoluting bulk gene expression profile with xCell

Figure S3 Validation of neutrophil activation makers and commonly used markers of psoriasis in Figure 1C,H, with the public datasets

Figure S4 The patterns of WGCNA

Figure S5 Ranks of regulators with gene regulatory network inferring for *core network*

Supporting Information

## Data Availability

The skin transcriptome data for validation is available in the GEO database (GSE186063).
