# Supplementary figures and images for: Multi‐omics integration reveals a core network involved in host defence and hyperkeratinization in psoriasis
Source: Clin Transl Med. 2022 Dec 19;12(12):e976. doi: 10.1002/ctm2.976 (PMC9763538; doi:10.1002/ctm2.976)

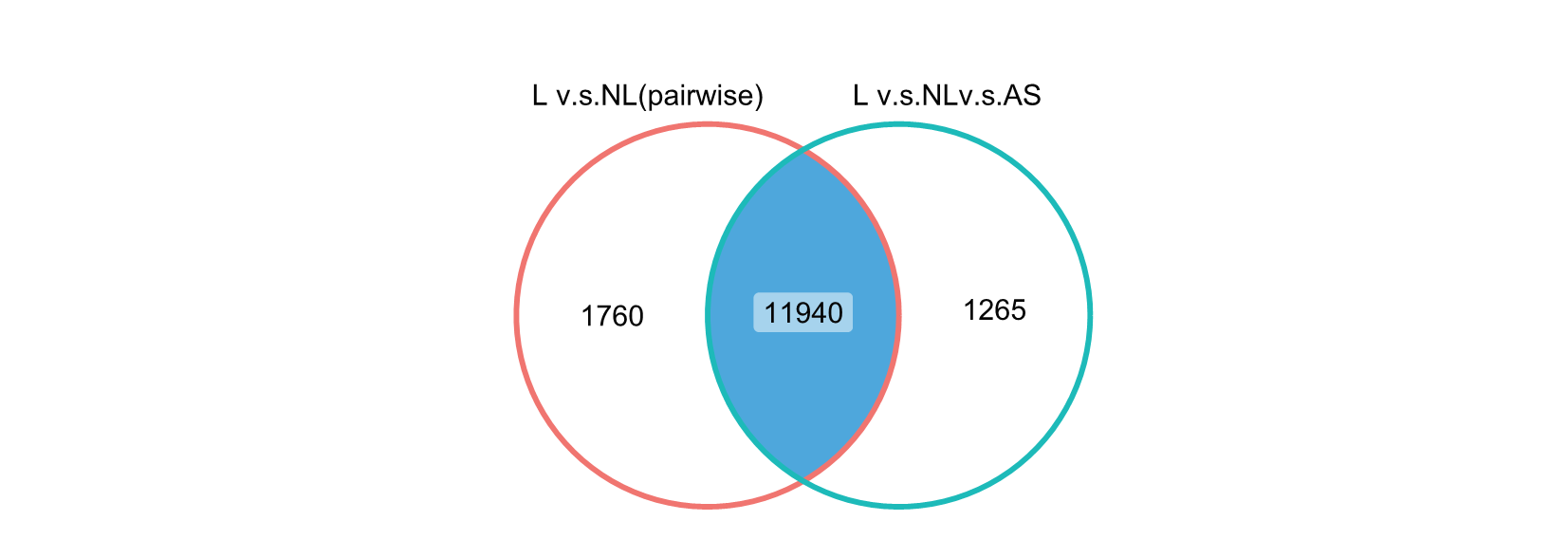

Supplement: Supplementary file 1 — Figure S1 Venn diagram showed the overlap of DEGs across the L versus NL pairwise comparison and L versus NL versus AS comparison. [file CTM2-12-e976-s004.tiff]

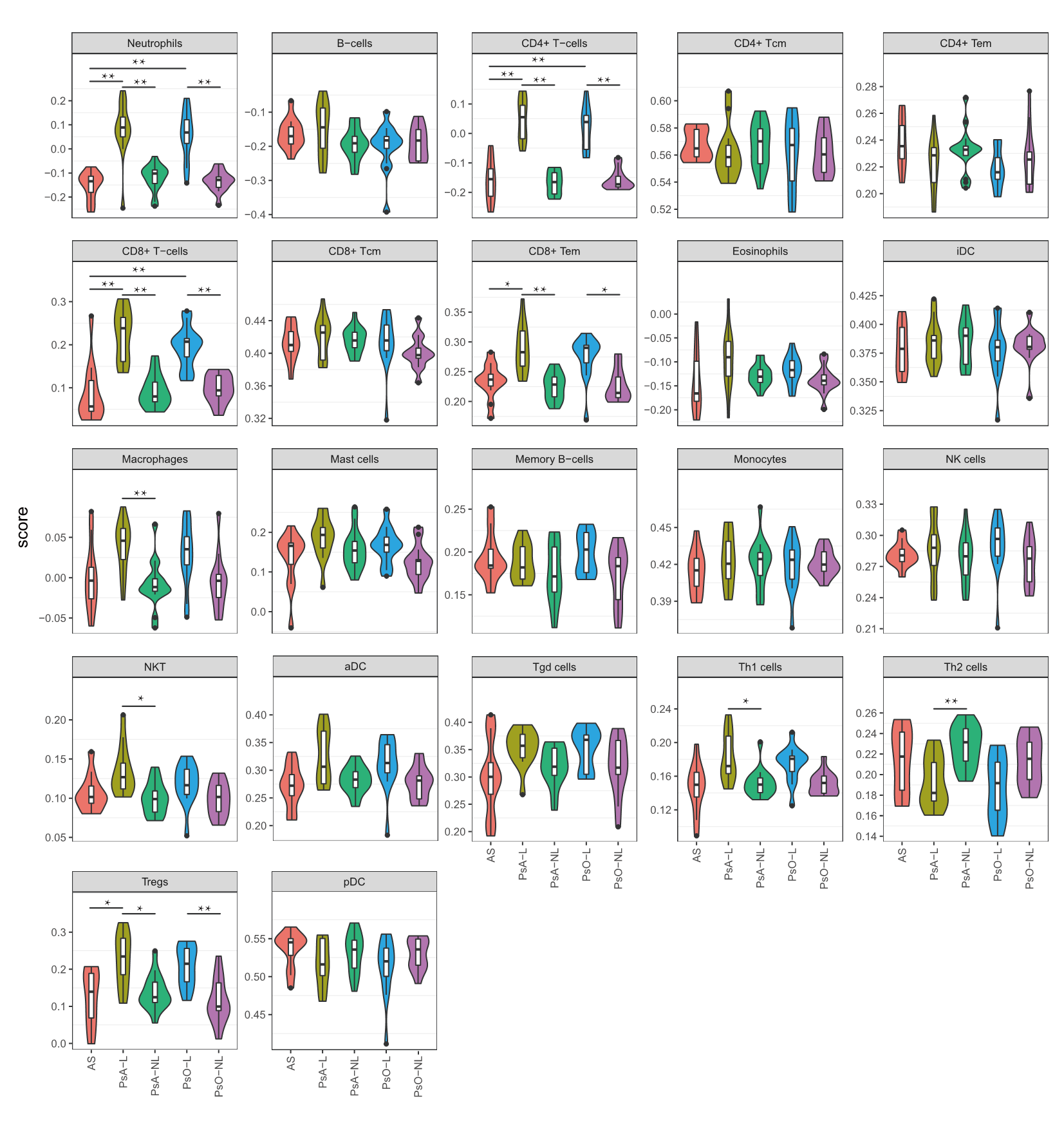

Supplement: Supplementary file 2 — Figure S2 Infiltration score of immune cells in skin predicted by deconvoluting bulk gene expression profile with xCell [file CTM2-12-e976-s002.tiff]

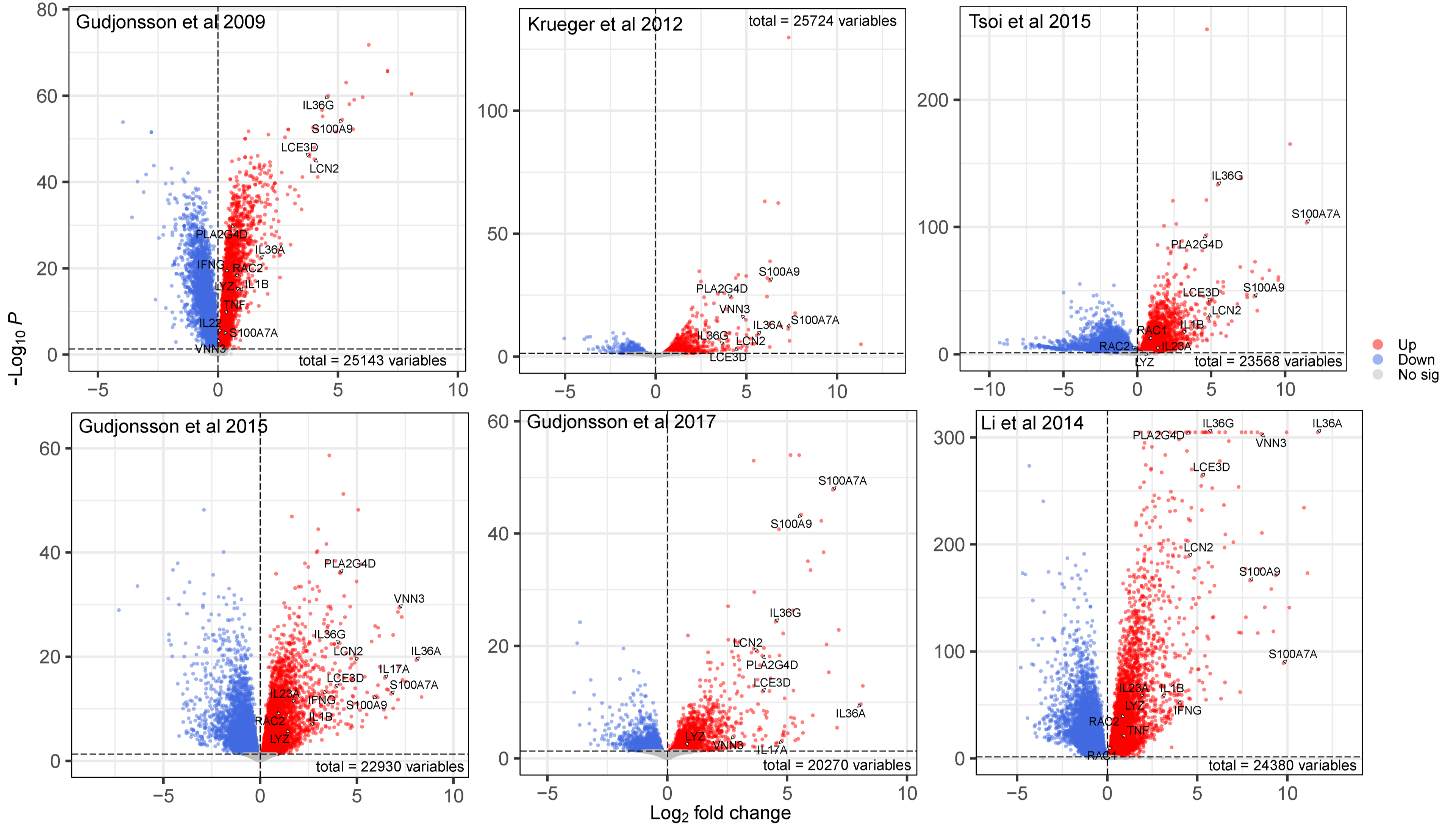

Supplement: Supplementary file 3 — Figure S3 Validation of neutrophil activation makers and commonly used markers of psoriasis in Figure 1C,H, with the public datasets [file CTM2-12-e976-s001.tiff]

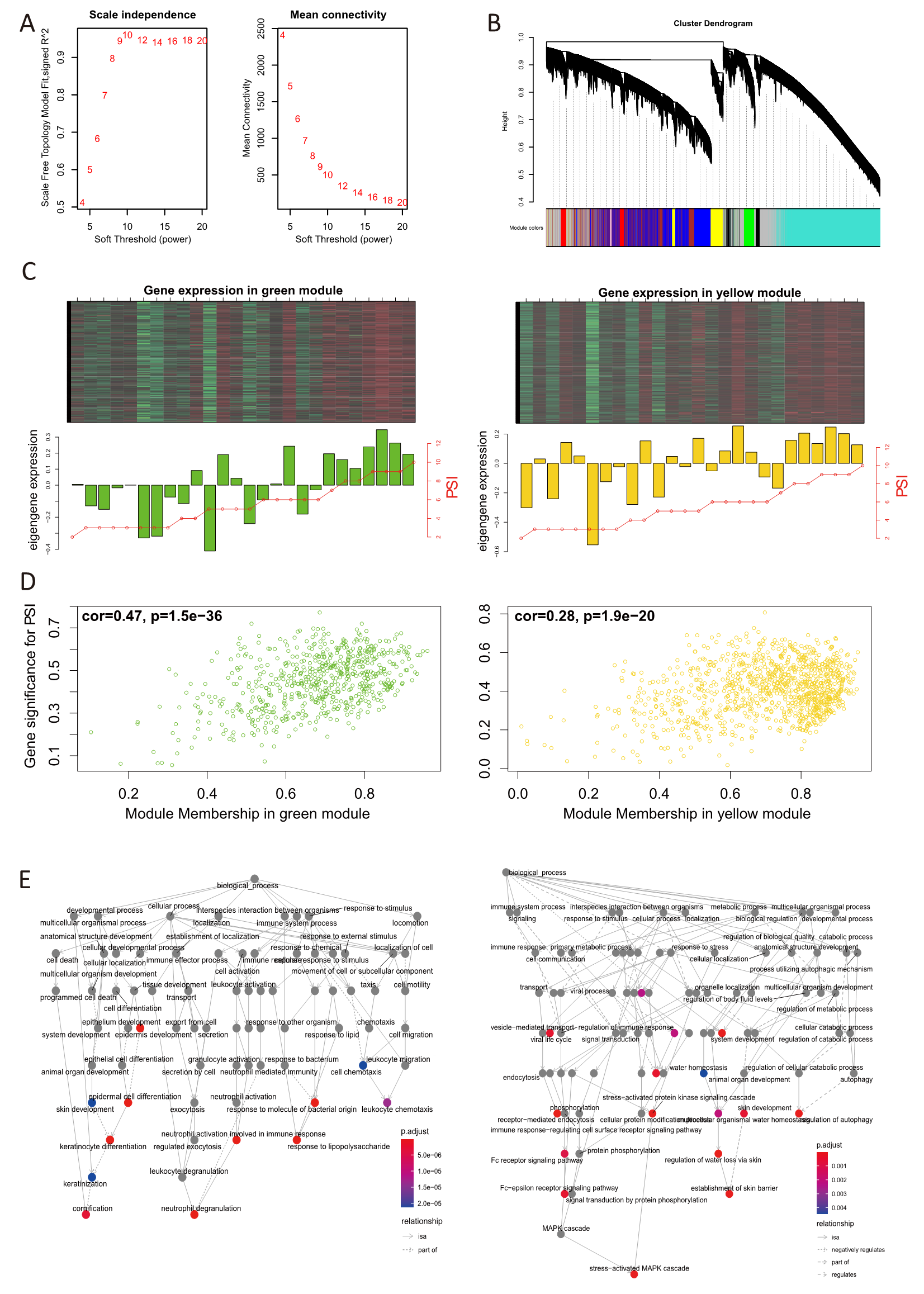

Supplement: Supplementary file 4 — Figure S4 The patterns of WGCNA [file CTM2-12-e976-s003.tiff]

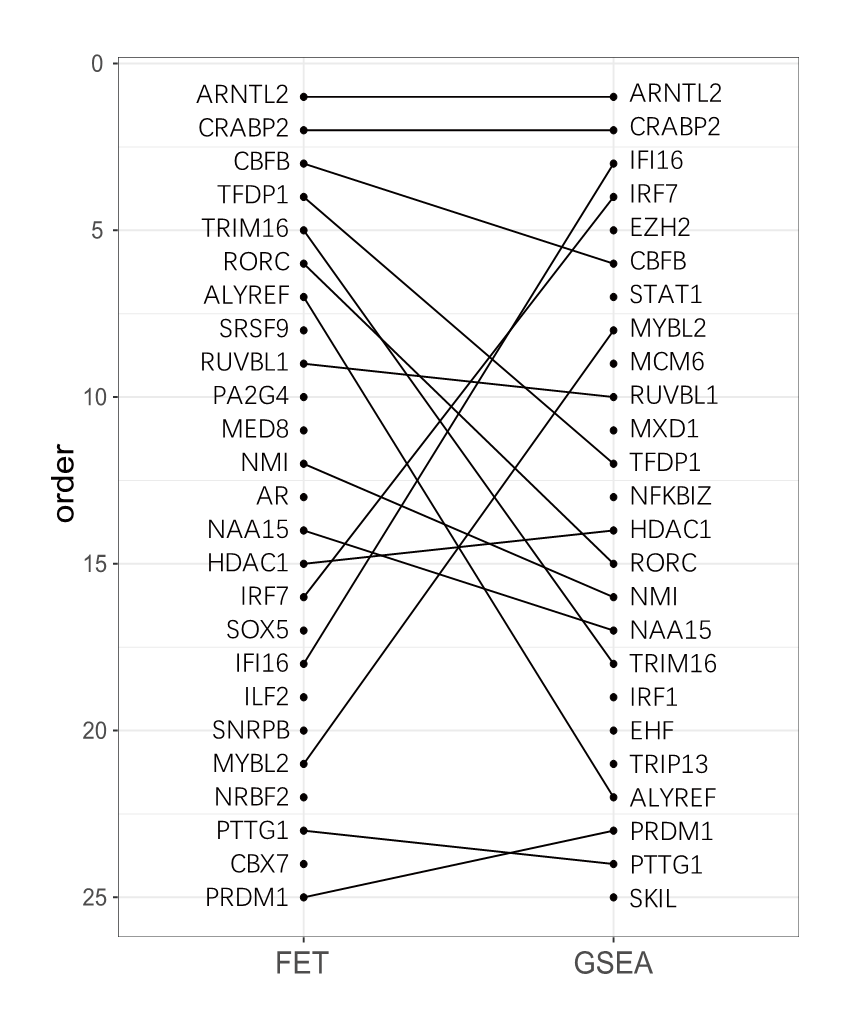

Supplement: Supplementary file 5 — Figure S5 Ranks of regulators with gene regulatory network inferring for core network [file CTM2-12-e976-s006.tiff]
